# Association of *GSTM1* and *GSTT1* Deletion Variants with Susceptibility to Behçet’s Disease in a Saudi Population: A Case Control Study

**DOI:** 10.3390/ijms27219568

**Published:** 2026-10-26

**Authors:** Mohammad Mustafa, Sarah Alkathiri, Seham Alrashidi, Shoug F. Algoblan, Fahdah Alokaily

**Affiliations:** 1 Department of Molecular Biology and Genetics, Research and Innovation Institute, Ministry of Defense Health Services, Riyadh 12372, Saudi Arabia; khan.mdmustafa@gmail.com; 2 Department of Medicine, King Faisal Specialist Hospital and Research Centre, Riyadh 12372, Saudi Arabia; sarahalkathiri98@gmail.com; 3 Division of Rheumatology, Department of Medicine, Prince Sultan Military Medical City, Riyadh 12372, Saudi Arabia; salrashidi@psmmc.med.sa (S.A.); s-f-algoblan@hotmail.com (S.F.A.)

**Keywords:** Behçet’s disease, multiplex PCR, glutathione S-transferase, null genotype, Saudi population

## Abstract

Behçet’s disease (BD) is a chronic multisystem inflammatory disorder in which genetic susceptibility and oxidative stress may contribute to disease pathogenesis. Glutathione S-transferase (GST) enzymes play an important role in cellular antioxidant defense. Deletion variants of the *GSTM1* and *GSTT1* result in loss of enzyme activity and may influence susceptibility to BD. A case control study was conducted including 79 patients with BD (*n* = 79) and 100 controls (*n* = 100) from Saudi Arabia. Genotyping of *GSTM1* and *GSTT1* deletion variants was performed using multiplex polymerase chain reaction. Genotype frequencies were compared using odds ratios (ORs) and 95% confidence intervals (CIs). The frequency of *GSTM1*− null genotype was significantly more frequent in BD cases than in controls (OR = 2.11, 95% CI = 1.15–3.85; *p* = 0.016; adjusted OR = 2.51, 95% CI = 1.24–5.07; *p* = 0.010; corrected *p* = 0.021). The *GSTT1*− null genotype was also more frequent (OR = 3.10, 95% CI = 1.63–5.89; *p* = 0.001; adjusted OR = 2.25, 95% CI = 1.08–4.68; *p* = 0.030), although this association did not remain significant after Bonferroni correction (corrected *p* = 0.060). Combined genotype analysis showed a significant association for *GSTM1*−/*GSTT1*− (OR = 6.90, 95% CI = 2.65–17.98; *p* < 0.001; adjusted OR = 6.35, 95% CI = 1.89–21.35; *p* = 0.003) compared with the *GSTM1*+/*GSTT1*+ genotype, which remained significant after Bonferroni correction (corrected *p* = 0.008). Stratified analyses did not demonstrate a clear association between *GST* deletion variants and disease activity or gender. The present study suggests that the *GSTM1*− genotype and the combined *GSTM1*−/*GSTT1*− genotype are significantly associated with susceptibility to BD in the Saudi population, independent of age and sex.

## 1. Introduction

Behçet’s disease (BD) is a chronic, relapsing, multisystem inflammatory disorder characterized by recurrent mucocutaneous lesions and variable systemic involvement. Although its exact etiology remains unknown, current evidence suggests that BD may result from a complex interplay of genetic susceptibility, immune dysregulation, and environmental factors [1]. The prevalence of BD varies widely worldwide, with the highest rates reported in Turkey (approximately 20–420 per 100,000) [2], followed by intermediate prevalence in Middle Eastern and Asian countries, including Saudi Arabia [3], and much lower rates in Western populations [4]. Recent evidence indicates that BD affects males and females at nearly equal frequencies, although a male predominance persists in some Arab populations [5]. Nevertheless, females more commonly present with arthritis and genital ulcers, whereas males more frequently develop ocular and central nervous system manifestations [6]. The profound clinical heterogeneity of BD and the episodic development of lesions across different organs and time points pose significant challenges for early diagnosis and disease management [7]. Although genetic susceptibility, environmental triggers, infectious agents, and immune dysregulation have all been implicated in disease pathogenesis, the precise mechanisms underlying BD remain incompletely understood. Several genes have been implicated in susceptibility to Behçet’s disease, with HLA-B*51 demonstrating the strongest and most consistently replicated genetic association across diverse ethnic populations, including the Saudi population [8,9,10,11]. However, genetic factors alone cannot fully account for the development of BD. Oxidative stress is increasingly recognized as a key contributor to the pathogenesis of BD, with excessive production of reactive oxygen species (ROS) promoting endothelial damage [12], lipid peroxidation [13], DNA damage [14], and dysregulated inflammatory responses [15] and chronic inflammation [16]. Recent studies have confirmed increased oxidative stress in BD, characterized by elevated malondialdehyde and nitric oxide levels and reduced antioxidant defenses, including glutathione, superoxide dismutase, catalase, and total antioxidant capacity, supporting the role of oxidant–antioxidant imbalance in disease pathogenesis [17,18]. Glutathione S-transferase (GST) enzymes, particularly GSTM1 and GSTT1, are important antioxidants involved in ROS detoxification. Common deletion variants in these genes result in loss of enzyme activity and may increase susceptibility to oxidative damage [19]. Therefore, *GSTM1* and *GSTT1* null genotypes have emerged as plausible genetic risk factors for BD.

Previous studies have reported significant associations between *GSTM1* and *GSTT1* deletion variants and susceptibility to BD, particularly in Turkish populations [20,21]. Moreover, these deletion variants have been implicated in the risk of several inflammatory and autoimmune disorders, including among Saudis [22,23,24]. However, no study has yet examined their association with BD in the Saudi population. In Saudi Arabia, the notably high prevalence of consanguineous marriage (~61%) [25] may contribute to a higher frequency of null genotypes such as those of *GSTM1* and *GSTT1* [26,27]. Notably, the frequencies of *GSTM1* and *GSTT1* deletion variants vary considerably among different ethnic and geographic populations, which may influence their contribution to disease susceptibility. These population-specific genetic factors, together with the high prevalence of consanguineous marriage, underscore the importance of directly investigating *GSTM1* and *GSTT1* deletion variants in Saudi patients with BD, as such variants may improve our understanding of the genetic determinants underlying disease susceptibility.

Therefore, the present study evaluated the association of *GSTM1* and *GSTT1* deletion variants, both individually and in combination, with the risk of BD in a Saudi population.

## 2. Results

The demographic analysis revealed that the BD group consisted of 54 (68.35%) males and 25 females (31.65%). The age of patients with BD ranged from 20 to 64 years (mean ± SD: 40.88 ± 8.62 years), while that of the control subjects ranged from 20 to 60 years (32.68 ± 7.42 years). Regarding clinical manifestations, all patients presented with oral ulcers. Other common features included genital ulcers (81.01%), ocular involvement (70.88%), musculoskeletal manifestations (67.08%), cutaneous involvement (60.75%), gastrointestinal involvement (35.44%), and neurological manifestations (22.78%), as shown in Table 1.

The frequencies of *GSTM1* and *GSTT1* deletion variants were analyzed to investigate their association with BD in the Saudi population. The frequencies of the *GSTM1*− and *GSTT1*− null genotypes were 50/79 (63.29%) and 38/79 (48.10%), respectively, among BD patients, compared with 45/100 (45.0%) and 23/100 (23.0%), respectively, in the control group (Table 2).

Comparison of genotype frequencies between cases and controls revealed that the *GSTM1*− null genotype was significantly associated with an increased risk of BD (OR = 2.11, 95% CI = 1.15–3.85, *p* = 0.016; adjusted OR = 2.51, 95% CI = 1.24–5.07, *p* = 0.010; corrected *p* = 0.021). The *GSTT1*− null genotype showed a comparable association (OR = 3.10, 95% CI = 1.63–5.89, *p* = 0.001; adjusted OR = 2.25, 95% CI = 1.08–4.68, *p* = 0.030), which did not retain significance after Bonferroni correction (corrected *p* = 0.060), as shown in Table 2. To further evaluate the combined effects of these deletion variants, four possible *GSTM1*/*GSTT1* genotype combinations were analyzed (Table 3). The frequency of the *GSTM1*+/*GSTT1*+ genotype was lower (14/79, 17.72%) among BD patients than controls (42/100, 42.0%). In contrast, the frequencies of the *GSTM1*−/*GSTT1*+, *GSTM1*+/*GSTT1*−, and *GSTM1*−/*GSTT1*− combined null genotype were higher in patients [27/79 (34.18%), 15/79 (18.99%), and 23/79 (29.11%), respectively] than in controls [35/100 (35.0%), 13/100 (13.0%), and 10/100 (10.0%), respectively] (Table 3). Using the *GSTM1*+/*GSTT1*+ genotype as the reference category, combined genotype analysis demonstrated a significant association for the *GSTM1*−/*GSTT1*− null genotype (OR = 6.90, 95% CI = 2.65–17.98; *p* < 0.001; adjusted OR = 6.35, 95% CI = 1.89–21.35; *p* = 0.003; corrected *p* = 0.008), indicating an increased risk of BD among carriers of this genotypic combination; the *GSTM1*−/*GSTT1*+ and *GSTM1*+/*GSTT1*− genotypes were not significant after Bonferroni correction (corrected *p* = 0.130 and 0.437, respectively) (Table 3). Genotype frequencies were compared between patients with active and inactive BD to explore whether the distribution of *GSTM1* and *GSTT1* deletion variants differed according to disease activity status; no significant differences were observed between the groups (Table 4). Likewise, analysis based on gender revealed no significant association between the *GSTM1* deletion variant (OR = 0.74, 95% CI = 0.27–2.02, *p* = 0.555) or the *GSTT1* deletion variant (OR = 1.27, 95% CI = 0.49–3.30, *p* = 0.620) and BD susceptibility (Table 5). The frequency of *GSTM1*, *GSTT1*, and combined *GSTM1*/*GSTT1* null genotypes varied considerably across different ethnic populations. The prevalence of the *GSTM1* null genotype ranged from 27.8% in Cameroonians to 63.4% in Tunisians, while the *GSTT1* null genotype ranged from 17.3% in Turkish population to 54.3% in Koreans. The combined *GSTM1*/*GSTT1* null genotype was highest among Koreans (29.1%) and lowest among the Indian population (9%), highlighting substantial inter-population variability in *GST* gene deletion frequencies (Table 6).

## 3. Discussion

The present study investigated the association of *GSTM1* and *GSTT1* deletion variants with susceptibility to BD in a Saudi population. When the frequencies of the *GSTM1*− and *GSTT1*− genotypes were compared between BD patients and controls, both the *GSTM1*− and *GSTT1*− null genotypes were found to be more prevalent among patients, indicating an association between these deletion variants to BD in the Saudi population. These findings are consistent with those reported by Al-Musawi et al. (2020) in an Iraqi population, who reported that *GSTT1*− null genotype was significantly more frequent among BD patients [41]. In another study, *GSTT1*− null genotype showed a significant association (OR = 2.74, *p* = 0.012) with an increased risk of venous insufficiency, a recognized vascular manifestation of BD (20). In our cohort, the *GSTT1*− null genotype was also more frequent among BD patients, although this association did not withstand correction for multiple comparisons (corrected *p* = 0.060). The frequency of the *GSTM1*− null genotype was likewise significantly higher and remained significant after Bonferroni correction (corrected *p* = 0.021). Nevertheless, the collective evidence suggests that *GST* deletion variants may contribute to BD susceptibility across different ethnic populations. Given the role of *GST* enzymes in cellular detoxification and protection against oxidative stress, these associations may reflect a contribution of *GSTM1* and *GSTT1* deficiencies to disease susceptibility.

This study was also designed to investigate the possible association of combined genotypes *GSTM1* and *GSTT1* with BD. We found *GSTM1*+/*GSTT1*+ was higher in controls, while the *GSTM1*−/*GSTT1*− null genotype was significantly associated with an increased risk of BD in the Saudi population (*p* < 0.001; adjusted *p* = 0.003; corrected *p* = 0.008) (Table 3). These findings suggest that the *GSTM1*+/*GSTT1*+ genotype is less frequent among BD patients, whereas the *GSTM1*+/*GSTT1*− and *GSTM1−/GSTT1*+ genotype did not show a significant association with disease risk after Bonferroni correction. Collectively, the observed associations indicate that *GSTM1*− deficient genotype combinations, particularly the *GSTM1*−/*GSTT1*− genotype, may contribute to increased susceptibility to BD in this Saudi cohort. However, these findings should be interpreted with caution and warrant validation in larger, independent populations. Consistent with our findings, Uzunoğlu et al. (2006) also reported a significant association between the *GSTM1*−/*GSTT1*− genotype and increased susceptibility to BD in a Turkish population [21]. The concordance of these findings across different populations further supports the role of *GST* deletion variants in BD susceptibility. As *GSTM1* and *GSTT1* are involved in the detoxification of reactive intermediates, the simultaneous absence of both enzymes may be associated with reduced antioxidant capacity and could partly explain the increased susceptibility observed in carriers of the combined null genotype [19,42,43]. The biological plausibility of these findings is supported by accumulating evidence implicating oxidative stress in BD pathogenesis. Recent studies have demonstrated elevated oxidative stress markers, along with reduced antioxidant defenses, in patients with BD compared with controls, and these oxidative stress markers have been shown to correlate with disease activity and severity [17,18]. Excessive ROS production by activated neutrophils has similarly been implicated in the endothelial dysfunction and persistent inflammation characteristic of BD [44]. Although associations between *GSTM1* and *GSTT1* null genotypes and oxidative stress markers have been reported in several complex human diseases [19,43,45], such relationships have not yet been evaluated in BD. As oxidative stress parameters were not assessed in the present study, this link remains inferential. However, it is consistent with the well-documented oxidant–antioxidant imbalance in BD and may plausibly contribute to the increased susceptibility associated with *GST* deficiency. Future studies integrating genetic and oxidative stress profiling are warranted to establish this relationship mechanistically. When the data were stratified according to disease activity, no significant differences in the distribution of *GSTM1* and *GSTT1* deletion variants were observed between patients with active and inactive BD (Table 4). To our knowledge, no previous study has examined the association of these variants with BD activity specifically, though genotype–severity associations have been reported for other *GST* related conditions; for example, the *GSTT1*− null genotype has been linked to disease severity and treatment response in aplastic anemia [46], suggesting this relationship may warrant further investigation in BD with larger cohorts. Given previous reports of sex-related differences in the clinical manifestations of BD, we performed sex-stratified analyses. No significant sex-specific associations were observed for *GSTM1* or *GSTT1* deletion variants; however, the relatively small number of female patients might have limited the power to detect such associations (Table 5). Although the observed associations support a role for *GSTM1* and *GSTT1* deficiencies in BD susceptibility, the biological effects of *GST* deletion variants may be context-dependent while reduced GST activity can impair the detoxification of reactive intermediates and exacerbate oxidative stress. GST-mediated metabolism may also generate reactive metabolites under certain conditions. Therefore, the influence of *GST* deletion variants on disease risk is likely complex, and both protective and deleterious effects should be considered when interpreting their contribution to BD pathogenesis.

To further interpret the observed association between *GST* deletion variants and BD susceptibility, it is important to consider the background distribution of these variants within the Saudi population and other ethnic groups. Population-specific differences in *GSTM1* and *GSTT1* deletion frequencies may influence the strength and consistency of genetic associations observed across studies.

In the present study, the null genotype frequencies of *GSTM1* (45.0%) and *GSTT1* (23.0%) observed in the control group were broadly similar to those previously reported in the Saudi population (56.1% and 20.7%, respectively), although the *GSTM1*-frequency was somewhat lower, which may reflect regional variation within Saudi Arabia [28]. Similar *GSTM1*− null genotype frequencies have also been reported in other Arab populations, including Bahrain, Lebanon, and Egypt, although a higher frequency has been observed in Tunisia [29,30]. In contrast, the *GSTT1*− null genotype appeared to occur less frequently in Saudis than in several neighboring Arab populations [29,30]. A comparable pattern of inter-population variation was also evident when the Saudi data were compared with Caucasian, Asian, and African populations. While *GSTM1*− null genotype frequencies were generally similar to those reported in Italy, the United Kingdom, and Spain, greater variability was observed for *GSTT1*− null genotype frequencies [38,39]. Overall, these findings demonstrate considerable ethnic and geographic differences in the distribution of *GST* deletion variants (Table 6). Such variation likely reflects differences in population genetic background, demographic history, and environmental exposures, highlighting the importance of using ethnically matched controls in genetic association studies.

Despite these findings, several limitations should be considered. Functional assessments of *GST* enzyme activity and oxidative stress biomarkers were not performed; therefore, the biological mechanisms underlying the observed associations could not be directly evaluated. In addition, the potential contributions of other genetic factors, environmental exposures, and gene-environment interactions were not examined. Furthermore, the multiplex PCR method used in this study distinguished only between the presence and absence of *GSTM1* and *GSTT1* genotypes and could not differentiate heterozygous (+/−) from homozygous present (+/+) genotypes; therefore, gene-dose effects, copy number variations, and inheritance models could not be assessed. The study sample was limited to eligible patients with BD available during the study period, which may have affected the generalizability of the findings, and the absence of an a priori sample size or power analysis raises the possibility that the study was underpowered to detect modest genotype-disease associations. In addition, disease activity was classified clinically at a single time point rather than with a validated activity index, which may have led to misclassification, given the relapsing remitting course of BD. Consequently, larger studies incorporating functional biomarkers, comprehensive genetic analyses, and environmental risk factors are needed to further elucidate the role of *GST* variants in BD susceptibility and pathogenesis.

## 4. Materials and Methods

The present study includes a total number of 79 BD patients and 100 controls recruited from Division of Rheumatology, Prince Sultan Military Medical City (PSMMC), Riyadh, Saudi Arabia. The study was approved by Institutional Review Board Research Ethics Committee (REC) Approval No. REC-457-02.04.2019, PSMMC. Written informed consent was obtained from all participants prior to sample collection. Controls were recruited from the same hospital population as the cases and included individuals attending for routine visits (health check-ups, pre-employment or pre-marital screening, vaccination visits, and blood-donor screening), non-inflammatory conditions (headache and functional dyspepsia), or mechanical musculoskeletal complaints (non-specific low back or neck pain and minor sprains/strains). Individuals with a history of BD, autoimmune disorders, chronic inflammatory diseases, or other significant systemic illnesses, as determined through clinical history and review of medical records, were excluded from the study. No additional laboratory screening was performed specifically for study enrollment.

The diagnosis of BD was based on the International Study Group (ISG 1990) criteria. Moreover, the principles of the Declaration of Helsinki were implemented in completing the present study. Patients were examined by trained physicians for clinical characteristics such as oral ulcers, genital ulcers, eye inflammation, musculoskeletal, cutaneous, gastrointestinal, nervous, respiratory, cardiovascular, and vascular lesions. Disease activity was assessed clinically at the time of recruitment; patients with active mucocutaneous, ocular, vascular, neurological, or other systemic manifestations documented on examination were classified as having active BD, and those without such manifestations as having inactive disease. A standardized activity index, such as the Behçet’s Disease Current Activity form, was not applied.

### 4.1. Genomic DNA Extraction

Genomic DNA for genotyping was extracted from whole blood using the QIAamp DNA Mini Kit (Qiagen, Venlo, Limburg, The Netherlands) according to the manufacturer’s instructions. Venous blood (3 mL) was collected from each participant into EDTA-coated tubes, transported to the laboratory in an icebox, processed, and stored at −80 °C until genomic DNA extraction. The extracted DNA was dissolved in nuclease free water, and its quality and integrity were assessed by agarose gel electrophoresis. DNA concentration and purity were determined using a NanoDrop 2000 spectrophotometer (Thermo Fisher Scientific Inc., Waltham, MA, USA).

### 4.2. Detection of GSTM1/GSTT1 Deletion Variants

Genotyping of *GSTM1* and *GSTT1* deletion variants was performed using multiplex PCR. Briefly, 50 ng of genomic DNA was amplified in a 25 μL reaction mixture containing 30 pmol of each *GSTM1* and *GSTT1* primer, 1.5 mM MgCl_2_, 200 μM dNTPs, 2.5 μL of 10× PCR buffer (500 mM KCl, 100 mM Tris-HCl, pH 9.0), and 1 U of Taq DNA polymerase. The primer sequences for *GSTM1* were 5′-GAA CTC CCT GAA AAG CTA AAG C-3′ (forward) and 5′-GTT GGG CTC AAA TAT ACG GTG G-3′ (reverse), while those for *GSTT1* were 5′-TTC CTT ACT GGT CCT CAC ATC TC-3′ (forward) and 5′-TCA CCG GAT CAT GGC CAG CA-3′ (reverse) [24]. Primers for the β-globin gene, used as an internal control to exclude false negative results, were 5′-GAA GAG CCA AGG ACA GGT AC-3′ (forward) and 5′-TGG TCT CCT TAA ACC TGT CTT G-3′ (reverse) [47].

**Figure 1:**
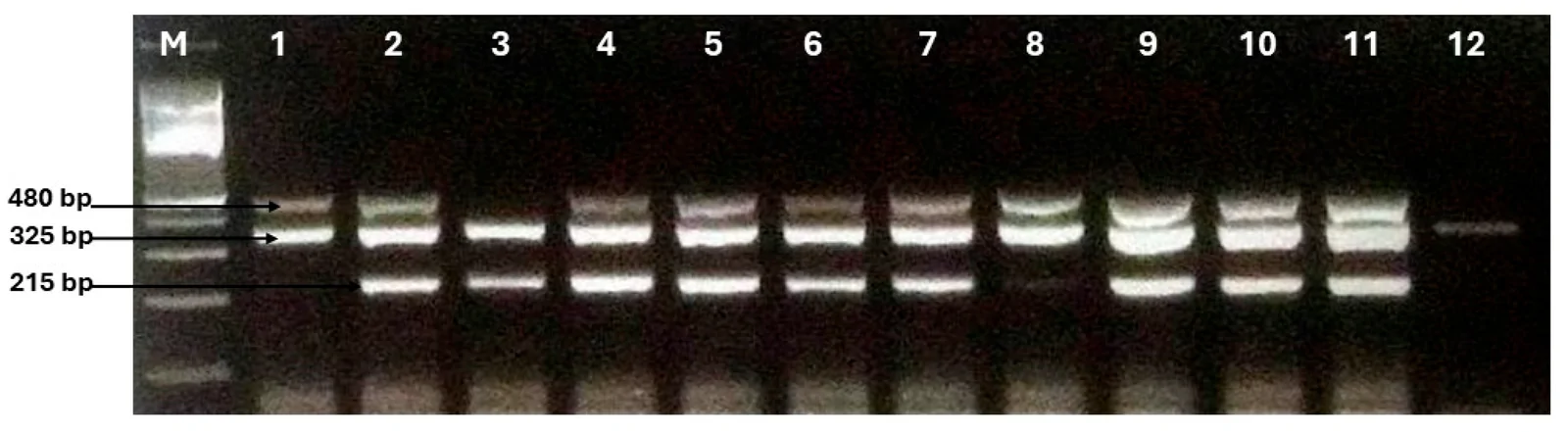
Gel picture showing *GSTM1* and *GSTT1* deletions. M-100 bp marker, Lane 2, 4, 5, 6, 7, 9, 10 and 11: *GSTM1* and *GSTT1* present, Lane 1 and 8: *GSTM1* deletion, Lane 3: *GSTT1* deletion and Lane 12: *GSTM1* and *GSTT1* both deletion.

PCR amplification was carried out with an initial denaturation step at 94 °C for 5 min, followed by 35 cycles of denaturation at 94 °C for 2 min, annealing at 63 °C for 1 min, and extension at 72 °C for 1 min, with a final extension at 72 °C for 10 min. The amplified products, including *GSTM1* (215 bp), β-globin (325 bp), and *GSTT1* (480 bp), were separated by electrophoresis on a 2.5% agarose gel stained with ethidium bromide (Sigma–Aldrich, St. Louis, MO, USA) and visualized under UV illumination. The absence of a *GSTM1* or *GSTT1* amplification product in the presence of the β-globin control band was interpreted as the respective null (*GSTM1*− or *GSTT1*−) genotype. The multiplex PCR assay detects only the presence or absence of the *GSTM1* and *GSTT1*; therefore, *GSTM1*+/*GSTT1*+ indicates the presence of at least one gene copy, whereas *GSTM1*−/*GSTT1*− denote homozygous deletions (Figure 1). To ensure genotyping accuracy, 19 randomly selected samples (10.6% of all samples), including both cases and controls, were independently re-genotyped by repeat PCR, with 100% concordance between the original and repeat results. The initial genotyping success rate was 95.0% (170/179), with nine samples yielding inconclusive results. Following repeat amplification, definitive genotypes were obtained for all samples, resulting in a final genotyping call rate of 100% (179/179). Samples lacking *GSTM1* or *GSTT1* amplification were considered null genotypes only in the presence of the β-globin control band; otherwise, the assay was repeated. Genotype interpretation was performed blinded to case-control status. 

### 4.3. Statistical Analysis

Statistical analyses were performed using SPSS software for Windows, version 21.0 (IBM Corp., Chicago, IL, USA). Differences in genotype frequencies between patients and controls were evaluated using binary logistic regression. The strength of the association was estimated by calculating crude and age- and sex-adjusted odds ratios (ORs) and 95% confidence intervals (CIs); each gene and each combined genotype was analyzed in a separate model. To account for multiple comparisons, Bonferroni correction was applied by multiplying the age and sex-adjusted *p* value by the number of comparisons performed within each analysis (two for single gene analyses and three for combined-genotype analyses). Comparisons between active and inactive BD patients, and between male and female patients, were also performed using binary logistic regression; these subgroup analyses were exploratory and are presented as crude ORs. A two-tailed *p* value of <0.05 was considered statistically significant.

## 5. Conclusions

The present study demonstrates that the *GSTM1*− null genotype and the combined *GSTM1*/*GSTT1* null genotype are significantly associated with susceptibility to BD in the Saudi population. However, larger studies incorporating functional analyses are needed to confirm these associations and further elucidate their biological and clinical significance.

## Figures and Tables

**Table 1:** Demographic and clinical manifestations of Behçet’s disease patients and controls.

Characteristic	BD Patients (n = 79)	Control (n = 100)
Sex		
Male	54 (68.35)	51 (51.00)
Female	25 (31.65)	49 (49.00)
Age (mean ± SD, years)	40.88 ± 8.62	32.68 ± 7.42
Clinical characteristics of BD patients		
Oral ulcers	79 (100.00)	NA
Genital ulcers	64 (81.01)	NA
Ocular involvement	56 (70.88)	NA
Musculoskeletal involvement	53 (67.08)	NA
Cutaneous involvement	48 (60.75)	NA
Gastrointestinal involvement	28 (35.44)	NA
Nervous system involvement	18 (22.78)	NA

Values in parentheses represent percentages. NA = Not available.

**Table 2:** Genotype distribution of *GSTM1* and *GSTT1* deletion variants in Behçet’s disease patients and controls.

Genotype	Case (n = 79)	Control (n = 100)	Crude OR (95% CI)	*p* Value	Adjusted OR (95% CI) ^a^	*p* Value	^b^ *p* Value Corr
*GSTM1*+	29 (36.71)	55 (55.00)	Reference	---	Reference	---	---
*GSTM1*−	50 (63.29)	45 (45.00)	2.11 (1.15–3.85)	0.016	2.51 (1.24–5.07)	0.010	0.021 *
*GSTT1*+	41 (51.90)	77 (77.00)	Reference	---	Reference	---	---
*GSTT1*−	38 (48.10)	23 (23.00)	3.10 (1.63–5.89)	0.001	2.25 (1.08–4.68)	0.030	0.060

^a^ Adjusted for age and sex. Crude and adjusted odds ratios (ORs) with 95% confidence intervals (CIs) were estimated using binary logistic regression, with *GSTM1* and *GSTT1* analyzed in separate models and the gene-present genotype as the reference category. ^b^ *p*_corr_ = Bonferroni-corrected *p* value for the two comparisons, obtained by multiplying the age- and sex-adjusted *p* value by 2 to account for the two genes tested (*GSTM1* and *GSTT1*). Confidence intervals were not adjusted for multiple comparisons. Statistical significance was determined based on *p*_corr_. Values in parentheses represent percentages. * *p*_corr_ < 0.05.

**Table 3:** Association of combined *GSTM1*/*GSTT1* genotype with Behçet’s Disease susceptibility.

Genotype	Case (n = 79)	Control (n = 100)	Crude OR (95% CI)	*p* Value	Adjusted OR (95% CI) ^a^	*p* Value	^b^ *p* Value Corr
*GSTM1*+/*GSTT1*+	14 (17.72)	42 (42.00)	Reference	---	Reference	---	---
*GSTM1*−/*GSTT1*+	27 (34.18)	35 (35.00)	2.31 (1.05–5.08)	0.036	2.43 (1.03–5.74)	0.043	0.130
*GSTM1*+/*GSTT1*−	15 (18.99)	13 (13.00)	3.46 (1.33–9.02)	0.011	2.23 (0.76–6.56)	0.146	0.437
*GSTM1*−/*GSTT1*−	23 (29.11)	10 (10.00)	6.90 (2.65–17.98)	<0.001 *	6.35 (1.89–21.35)	0.003 *	0.008 *

^a^ Adjusted for age and gender. Crude and adjusted odds ratios (ORs) with 95% confidence intervals (CIs) were estimated using binary logistic regression, with each combined genotype analyzed separately against the *GSTM1*+/*GSTT1*+ reference genotype. ^b^ *p*_corr_ = Bonferroni-corrected *p* value, calculated by multiplying the age- and gender adjusted *p* value by the number of comparisons, with each combined genotype compared against the *GSTM1*+/*GSTT1*+ reference genotype. Confidence intervals are unadjusted for multiple comparisons; statistical significance is based on *p*_corr_. Values in parentheses represent percentages. * *p*_corr_ < 0.05.

**Table 4:** Comparison of *GSTM1* and *GSTT1* genotypes frequencies between active and inactive BD patients.

Genotype	Active (n = 39)	Inactive (n = 40)	OR (95% CI)	*p* Value
*GSTM1*+	13 (33.33)	16 (40.00)	Reference	---
*GSTM1*−	26 (66.67)	24 (60.00)	1.33 (0.53–3.34)	0.539
*GSTT1*+	23 (58.97)	18 (45.00)	Reference	---
*GSTT1*−	16 (41.03)	22 (55.00)	0.57 (0.23–1.39)	0.215

Odds ratios (ORs) and 95% confidence intervals (CIs) were estimated using binary logistic regression to compare active and inactive patients. The gene-present genotype (*GSTM1*+ or *GSTT1*+) was used as the reference category. Values in parentheses represent percentages.

**Table 5:** Comparison of *GSTM1* and *GSTT1* genotypes frequencies between male and female patients with BD.

Genotype	Male (n = 54)	Female (n = 25)	OR (95% CI)	*p* Value
*GSTM1*+	21 (38.89)	8 (32.00)	Reference	---
*GSTM1*−	33 (61.11)	17 (68.00)	0.74 (0.27–2.02)	0.555
*GSTT1*+	27 (50.00)	14 (56.00)	Reference	---
*GSTT1*−	27 (50.00)	11 (44.00)	1.27 (0.49–3.30)	0.620

Odds ratios (ORs) with 95% confidence intervals (CIs) were estimated using binary logistic regression to compare male and female patients. The gene-present genotype (*GSTM1*+ or *GSTT1*+) was used as the reference category. Values in parentheses represent percentages.

**Table 6:** Frequency of *GSTM1*, *GSTT1*, and combined *GSTM1*/*GSTT1* null genotypes in different populations.

Population	*GSTM1*− (%)	*GSTT1*− (%)	*GSTM1*−/*GSTT1*− (%)
**Arab**			
Saudi Arabia [28]	56.1	20.7	11.0
Bahrain [29]	49.7	28.7	14.4
Lebanon [29]	52.5	37.6	16.3
Tunisia [29]	63.4	37.1	21.0
Egypt [30]	55.5	29.5	NR
**Asian**			
Turkey [31]	51.9	17.3	NR
Iran [32]	44.7	21.2	NR
India [33]	33.3	32	9
China [34]	36.4	38.8	20
Japan [35]	47.9	47.4	23.2
Korea [36]	53.8	54.3	29.1
**Caucasian**			
United Kingdom [37]	54.8	18.5	9.4
Spain [38]	42.4	19.2	NR
Italy [39]	49.2	28.3	NR
**African**			
Cameroon [39]	27.8	46.8	NR
Ivory Coast [40]	36.1	33.1	14.3
Ethiopia [39]	43.8	37.3	NR

NR = Not reported, figures in parenthesis show percentage values.

## Data Availability

The data supporting the findings of this study are available from the corresponding author upon reasonable request.
